# Epitaxial Growth of Bi_2_Se_3_ Infrared Transparent Conductive Film and Heterojunction Diode by Molecular Beam Epitaxy

**DOI:** 10.3389/fchem.2022.847972

**Published:** 2022-01-25

**Authors:** Ya-Hui Chuai, Chao Zhu, Dan Yue, Yu Bai

**Affiliations:** ^1^ Institute of Physics, Changchun University of Science and Technology, Changchun, China; ^2^ College of Electronical and Information Engineering, Changchun University of Science and Technology, Changchun, China

**Keywords:** thin film, Bi2Se3, optical property, electronic property, N-Bi2Se3/P-CuScO2 heterojunction

## Abstract

Epitaxial *n*-type infrared transparent conductive Bi_2_Se_3_ thin film was cultivated by molecular beam epitaxy (MBE) method on Al_2_O_3_ (001) substrate. The orientation between Bi_2_Se_3_ and the substrate is Bi_2_Se_3_(001)//Al_2_O_3_(1 $\bar{2}$ 10). Conducting mechanism ensued the small-polaron hopping mechanism, with an activation energy of 34 meV. The film demonstrates conductivity of *n*-type, and the resistivity is 7 × 10^−4^ Ωcm at room temperature. The Film exhibits an excellent carrier mobility of 1,015 cm^2^/Vs at room temperature and retains optical transparency in the near-infrared (>70%) and far-infrared (>85%) ranges. To the best of our knowledge, the Bi_2_Se_3_ film yields the best result in the realm of *n*-type Infrared transparent conductive thin films generated through either physical or chemical methods. To demonstrate the application of such films, we produced N-Bi_2_Se_3_/P-CuScO_2_ heterojunction diode device, the ∼3.3 V threshold voltage of which conformed fairly well with the CuScO_2_ bandgap value. The high optical transparency and conductivity of Bi_2_Se_3_ film make it very promising for optoelectronic applications, where a wide wavelength range from near-infrared to far-infrared is required.

## Introduction

Infrared transparent conductive film is widely used in military and civilian infrared detectors, such as Infrared guidance, Infrared imaging, Infrared detection and Infrared methane/CO detector etc. (Zhang et al., 2021), because of its remarkable optical transmittance in the Infrared light range and strong electromagnetic shielding ability. However, the traditional wide band gap oxide transparent conductive film such as ITO (Sn-doped In_2_O_3_) and AZO (Al-doped ZnO) (Jain et al., 2020; Khan et al., 2020), can only transmit visible light and near-infrared light, and has low transmittance in the mid- and far-infrared bands. At present, infrared detectors are developing in the direction of all-weather high sensitivity, such as dual-use day and night, wider infrared spectrum detection range (mid-to-far infrared band), and adaptability to complex electromagnetic interference signal environments (Zhang et al., 2020; Sizov et al., 2021). Therefore, it is particularly important to develop a wide-band infrared transparent conductive film.

Up to now, a few infrared transparent conductive films have been developed by researchers. T. Chen from Yale University reported a doped In_2_O_3_-based infrared transparent conductive film for the first time, which has a transmittance of 40% in the 2.5–12 μm band and a resistance of 30 Ω/□ (Chen et al., 1983). N. Lipkin prepared In_x_O_y_ thin films by using reactive ion evaporation and controlled oxygen defects, achieving a mid- and far-infrared transmittance of up to 72%, but the thin-film resistance reached 40 Ω/□ (Lipkin et al., 2001). E. Aydin reduced the resistance of the In_2_O_3_-based film to 18 Ω/□ through Zr doping by magnetron sputtering, while the transmission band is blue-shifted to the near-infrared region of 0.25–2.5 μm (Aydin et al., 2019). L. Johnson prepared Cu_x_Al_y_O_z_ and Cu_x_Cr_y_O_z_ amorphous films that deviate from the stoichiometric ratio by asymmetric bipolar pulsed DC magnetron sputtering method. The former has 70% transmittance in the mid-infrared band and the sheet resistance is 26 Ω/□, and the latter has a transmittance of 55% in the far infrared band (Johnson and Moran, 2001). Jicai Han prepared Ru-doped Y_2_O_3_ film on a ZnS substrate by plasma bombardment assisted magnetron sputtering, the transmittance of the film is 65% in the 3.5–12 μm infrared band, and the resistance is 3.36 × 10^2^ Ω/□ (Yang et al., 2013). The author has also developed a series of P-type doped CuFeO_2_ and CuScO_2_ based infrared transparent conductive films. The highest transmittance in the 0.78–5 μm near mid-infrared band is 90%, and the lowest resistivity is 2.25 × 10^3^ Ω/□ (Chuai et al., 2015a; Chuai et al., 2015b; Chuai et al., 2016a; Chuai et al., 2016b; Chuai et al., 2019). In summary, the classic design strategy of infrared transparent conductive film is mainly to prepare the wide band gap semiconductor oxide by element doping and stoichiometric deviation film preparation, and adjust the composition to improve the infrared transmittance and conductivity of the film at the same time. However, doping tends to cause a blue shift in the infrared transmission band of the oxide, and the film that deviates from the stoichiometric ratio has poor crystallinity and high resistance. Therefore, wide band gap semiconductor oxide is not an ideal mid- and far-infrared transparent conductive material.

Bismuth chalcogenides materials Bi_2_Se_3_ has gained attention due to its unique physical properties as a three-dimensional topological insulator, and potential applications in spintronics, optoelectronics and quantum computing (Xia et al., 2009; Qi and Zhang, 2011; Jash et al., 2020). Bi_2_Se_3_ has a body state (with an insulator band gap) and a surface state (without a band gap). The surface state can exist stably because of the protection of the time reversal symmetry. Therefore, it is externally conductive on the surface and insulated on the inside. Bi_2_Se_3_ is a typical V-VI compound semiconductor, belonging to the hexagonal crystal system, space group D5 3d (R3m), with a narrow band gap energy about 0.5 eV (Zhang et al., 2009). Bi_2_Se_3_ has a layered structure, each unit contains 5 atomic layers, oriented along the *Z* axis, and the stacking sequence is Se1-Bi1-Se2-Bi1′-Se1′, defined as 1 Quintuple Layer (QL), each QL The thickness is about 0.995 nm. There is a strong chemical bond between the two atomic layers inside a QL, while the bond between QLs is weak ---van der Waals force. The basic feature of the Bi_2_Se_3_ three-dimensional topological insulator is that there are four time-reversed symmetry points in the Brillouin zone of its surface state. Kramers degeneracy occurs at these points, thus forming Dirac cones. The apex of the Dirac cone is called the Dirac point, and the dispersion relationship between energy and momentum near the apex is linear (not quadratic). Due to the spin-coupling effect, the spin direction of the surface state is always perpendicular to the direction of momentum, so that electrons travel on the surface with low loss (or lossless) at a speed similar to photons (surface mobility μ_s_ ≈ 6000 cm^2^/Vs), the inside is in an insulator state (Hasan and Kane, 2010). Therefore, the band gap of Bi_2_Se_3_ yields a low free-carrier plasma oscillation cut-off frequency, and high transparency in the infrared (IR) range. Excellent surface mobility makes it also have high electrical conductivity which make it an ideal wide-band high infrared transmittance and conductivity film.

Though Bi_2_Se_3_ film can be fabricated by magnetron sputtering, chemical vapor deposition, and electrodeposition method. But still unable to prepare Bi_2_Se_3_ film with stoichiometric ratio. Because selenium (Se) is highly volatile, Bi_2_Se_3_ tends to form Se vacancies which act as donors to give a rather high carrier concentration and low carrier mobility (Richardella et al., 2010; Liu et al., 2015). During thin-film growth, when Se-atoms are lost to a greater extent at elevated substrate temperatures, pure phase Bi_2_Se_3_ film can barely survive, and the films obtained might present impure phases or turn into another phase all together. The high carrier concentration will blue shift the infrared transmission band, which will shorten the infrared transmission area. The low carrier mobility will cause the resistivity of the film to decrease. To counter this problem and achieve stoichiometric Bi_2_Se_3_ single crystal thin film with greater quality, we employed molecular beam epitaxy (MBE) to make the single crystal film at standard stoichiometric ratios.

In this letter, we use molecular beam epitaxy (MBE) to prepare high-quality Bi_2_Se_3_ single crystal thin film on Al_2_O_3_ (001) substrate. The main advantages of MBE are as follows: The film can grow at a low growth temperature and a slow growth rate, and it is easier to fine-tune the beam intensity, and timely adjust the composition of the film according to the change of the source, and single crystal films with a thickness of dozens of atomic layers can be prepared. Thus, the Se vacancy defect density in the thin film is reduced, and the body electron concentration is reduced. As far as we know, this is the first time that the infrared transmission characteristics of Bi_2_Se_3_ film is reported. We expect that the present results are greatly helpful for the practical usage of Bi_2_Se_3_ as novel wide band infrared transparent conductive film.

## Experiment and Calculation

### Preparation of Bi_2_Se_3_ Thin Film

Molecular beam epitaxy (MBE) was employed to prepare Bi_2_Se_3_ thin film on Al_2_O_3_ (001) substrate. Before film growth, the substrate was cleaned in UHV at 300°C for 15 min. Bi was sourced from high purity bismuth (Bi, 99.999%), and Se from selenium (Se, 99.999%). In order to obtain Bi and Se vapour, Bi and Se were put into the effusion tank for co-evaporation. The beam flux ratio of Bi and Se was 1:20, and the growth rate was ∼0.4 quintuple layer (QL) per minute. The total pressure was kept constant at 30 Pa. To enhance the crystalline property of the film, we adopted a two-step growth method. This method can increase the nucleation density of crystal grains on the substrate without causing tex-turing of the film (Park et al., 2016). In the first step, the substrate was heated to 170°C, and then deposited the initial 3–4 QLs of Bi_2_Se_3_ thin film. Then the obtained ultra-thin film was annealed at 300°C in H_2_ atmosphere for 30 min. In the second step, the substrate temperature was raised to 350°C at a rate of 4°C per minute. Then continue to grow to the predetermined thickness of the film. To assess the stability and antioxidant capacity of the Bi_2_Se_3_ film, all the tests were carried out after 2 weeks of air exposure.

### Characterization and Measurement

Conventional X-ray diffraction (XRD) technique was employed to represent the crystallographic orientation of the Bi_2_Se_3_ film on a Bruker D8 Advance X, Pert diffractometer (Cu-Ka: *λ* = 1.540 A). A scanning speed of 8° per min was chosen, ranging from 10° to 90°. X-ray photoelectron spectroscopy (XPS, ESCALAB 250) was used to determine the valence states of the elements. To detect the surface morphology of the film, feld emission scanning electron microscope (FE-SEM JSM-7500F) and atomic force microscope instrument (AFM Veeco DI-3100) were used. To further investigate the atomic arrangement of the film, a high-resolution transmission electron microscope (HRTEM, TEM 2010F) was used. To measure the optical properties of the film, such as transmission and absorption, a UV-vis-NIR spectrophotometer (Shimazu UV-3600PC) was used working in the wavelength range of 250–3,000 nm, plus a Fourier transform infrared spectrometer (FTIR) working in the range of 2.5–12 μm. To examine the electrical properties, a Hall-effect measurement system (ACCENT HL55OOPC) was introduced, the test range was between 90 and 300 K.

## Results and Discussion

### Structural and Chemical Valence Characteristics

**FIGURE 1:**
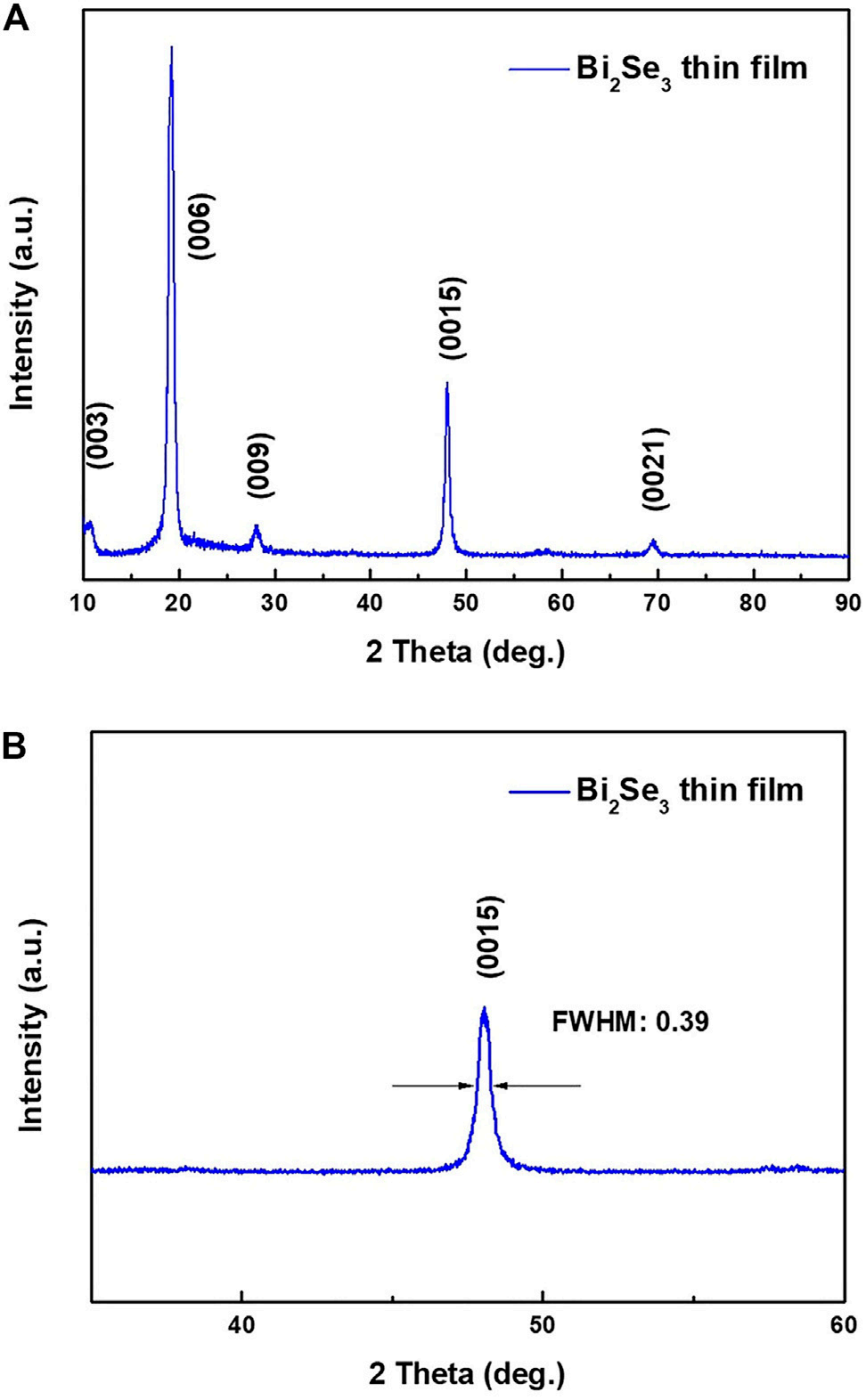
**(A)** XRD pattern of the Bi_2_Se_3_ single crystal film, **(B)** Zoomed in XRD pattern of the Bi_2_Se_3_ film at (0015) peak.

Figure 1A shows the XRD patterns of the resulting Bi_2_Se_3_ film. The only peaks observed in the scanned range are clearly defined and high intensity reflections [(003), (006), (009), (0015), (0021)]. The appearance of only (001) diffraction peaks indicates that the film is preferentially oriented along the *c*-axis perpendicular to the substrate surface. Further, the layered structures of Bi and Se atoms in the form of Se(1)-Bi-Se (2)-Bi-Se(1) are normal to the *c*-axis. The width of the half-maximum (FWHM) of the (0015) rocking curve (Figure 1B) is about 0.39°, indicating good crystallinity of the film. From the d-spacing’s of the (0015) peak, the lattice parameters of the Bi_2_Se_3_ film were determined as *a* = *b* = 4.138 (3) Å and *c* = 28.666 (2) Å , which conforms well to the reported standard card data file (No. 01-089-2008).

**FIGURE 2:**
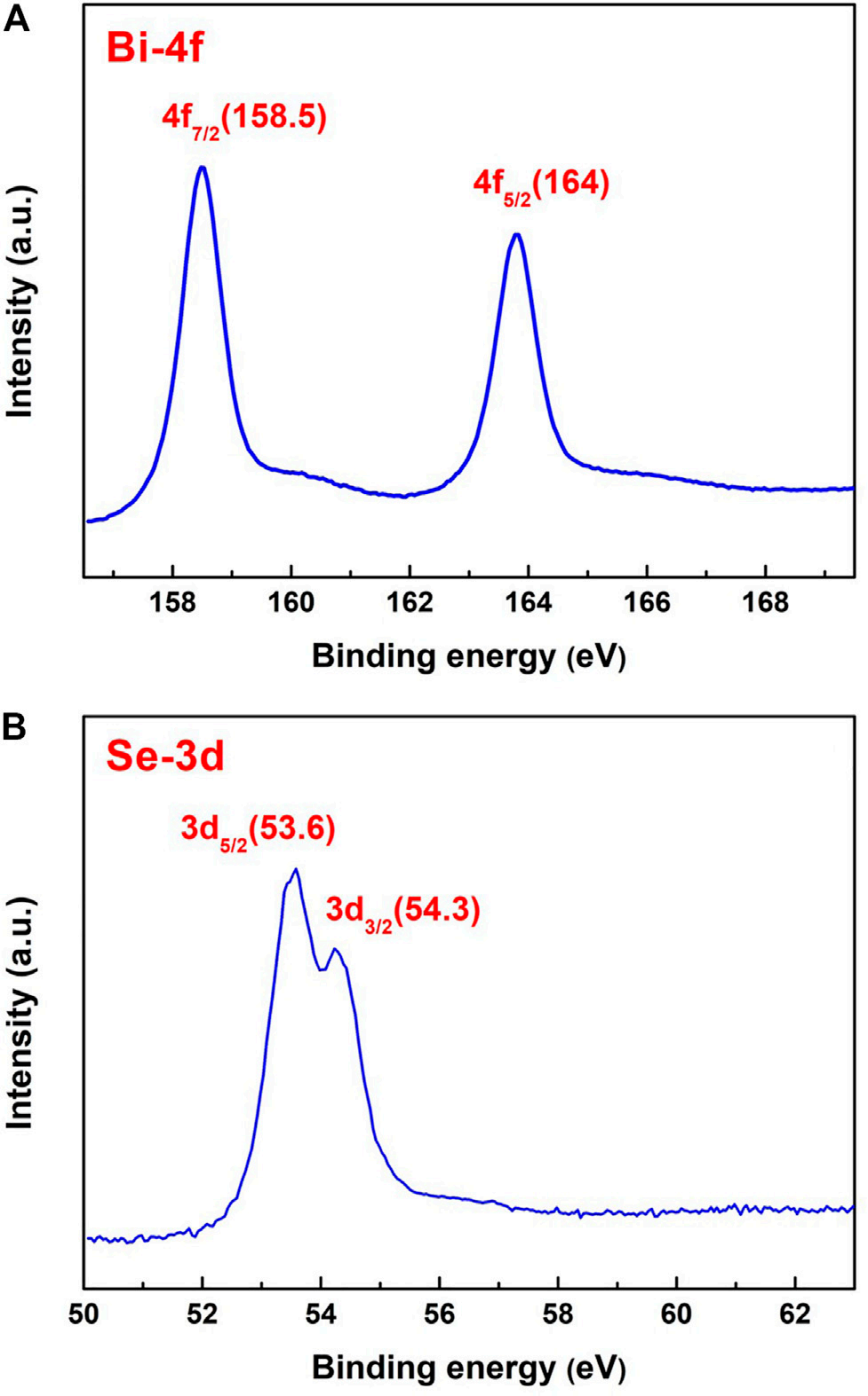
X-ray photoelectron spectra of Bi_2_Se_3_ thin film, **(A)** Bi-4f, **(B)** Se-3d.

Figure 2 shows the X-ray photoelectron spectra (XPS) of Bi-4f and Se-3d of Bi_2_Se_3_ thin film. In the Bi-4f spectrum, two distinct peaks Bi-4f_7/2_ = 158.5 eV and Bi-4f_5/2_ = 164 eV are observed, both are intense and with binding energy. This is consistent with the Bi₂O₃ (Bi^3+^) phase, which leads to the conclusion that the Bi ions are in the tervalent state. Figure 2B reveals two adjacent sharp peaks in the Se-3d_5/2_ spectrum at 53.6 and 54.3 eV on 3d_3/2_ peaks, respectively. Compare with the X-ray Photoelectron Spectroscopy Database of the National Institute of Standards and Technology (NIST), and it can be inferred that the valence state of the Se ions is −2. XPS analysis reveals the valence states of Bi and Se to be +3 and −2 in the Bi_2_Se_3_ film, which is conducive to the formation of a pure Bi_2_Se_3_ phase. The atomic content of Bi and Se on the surface of the film is 0.47 and 0.53 respectively. The deviation of the surface stoichiometric ratio is due to the lower vapor pressure of the Se element, so Se is prone to volatilization during the growth process, especially on the surface of the film.

### Morphologies and Microstructure of the Bi_2_Se_3_ Film

**FIGURE 3:**
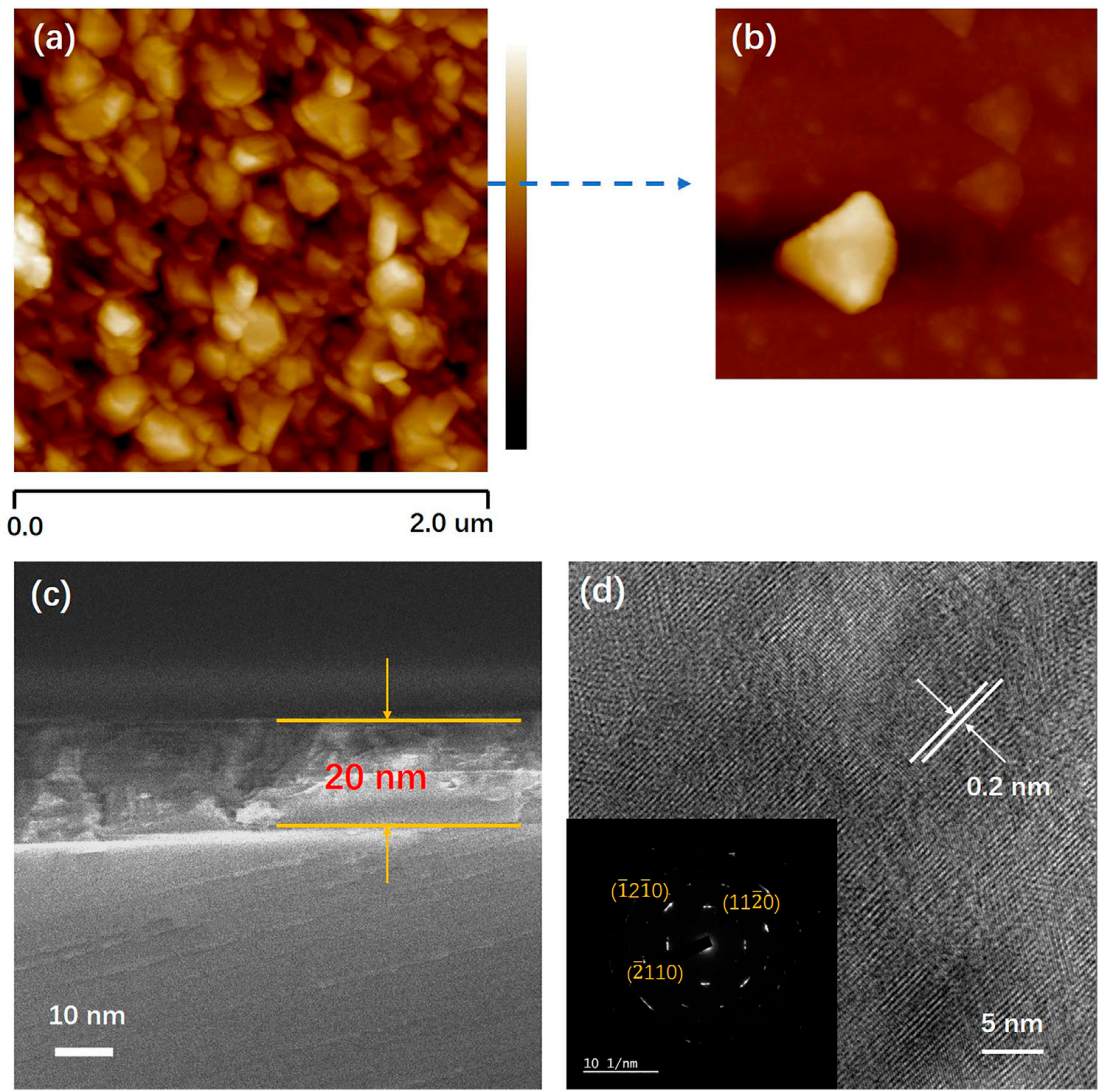
**(A)** 2 μm × 2 μm atomic force microscope images of the Bi_2_Se_3_ thin film, **(B)** Enlarged image of Bi_2_Se_3_ grain, **(C)** Cross-sectional SEM image of Bi_2_Se_3_ (001) thin film close to the interface of the substrate, **(D)** Surface HRTEM image and SAED pattern of Bi_2_Se_3_ thin film.

Figure 3A shows the film’s surface morphology atomic force microscope (AFM) image. It shows that the nano-particles is uniform and arrange orderly with an average size of 120 nm. As shown in Figure 3B, a higher-magnification view of the surface revealed triangular crystal facets and terrace structures of the Bi_2_Se_3_ thin film. The root-mean-square (RMS) roughness value for the 2 μm × 2 μm area is 0.9 nm. The thickness of Bi_2_Se_3_ film is shown in Figure 3C. The image indicates that the Bi_2_Se_3_ film thickness is uniform and tightly bonded to the substrate. No lattice defects such as threading dislocations are observed at the interface. In Figure 3D, the interplanar d spacing between lattice fringes is measured to be 0.2 nm, in conformance with the d-spacing of the (11 $\bar{2}$ 0) planes in the pdf file No. 01-089- 2008. Selected area electron diffraction pattern (SAED) of Bi_2_Se_3_ film shows three different diffraction spacings ([11 $\bar{2}$ 0] [ $\bar{1}$ 2 $\bar{1}$ 0] and [ $\bar{2}$ 110]) indexed as a 6-fold symmetric [001] zone axis pattern, conforming to the layered structure along the *c*-axis orientation. The above conclusions indicate that the Bi_2_Se_3_ film is a single crystal film, and it preferentially grows along the *c*-axis direction.

### Optical Properties of Bi_2_Se_3_ Thin Film

**FIGURE 4:**
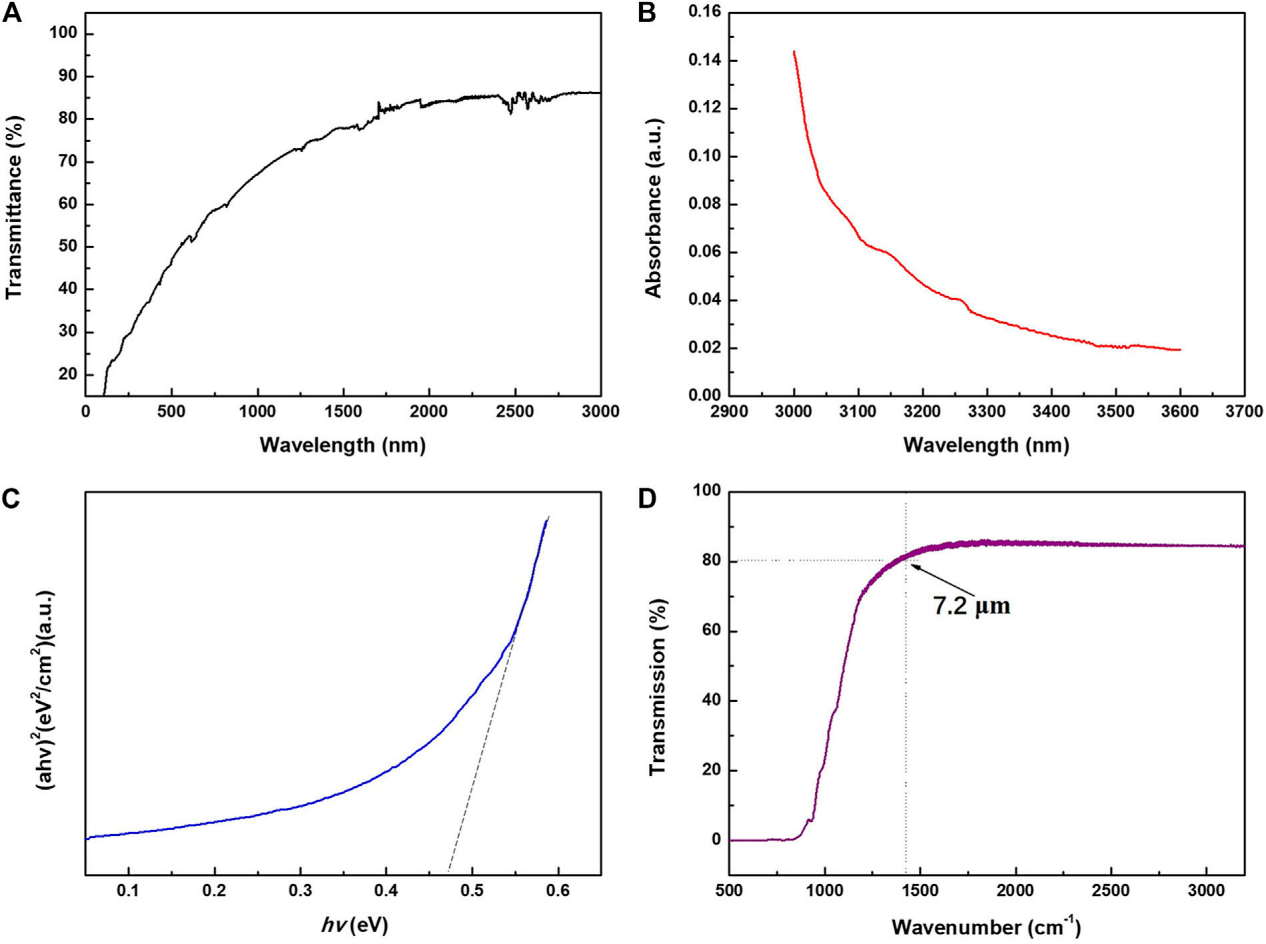
**(A)** Optical transmission spectrum of Bi_2_Se_3_ thin film, **(B)** Absorption spectrum of Bi_2_Se_3_ thin film, **(C)** (*ahv*)^2^ versus *hv* plot, and **(D)** Infrared transmission spectra of Bi_2_Se_3_ thin film.

Figure 4A gives the optical transmission spectrum of the Bi_2_Se_3_ film in visible light-near infrared band. All the measured data have been deducted from the influence of the substrate. The film exhibits a transmittance of 50–70% in the entire visible light band, and a higher transmittance of 70–85% in the near-infrared band. Figure 4B gives the absorption spectrum of Bi_2_Se_3_ thin film. At 3,000 nm wavelength stands a sharp absorption edge. It is the fundamental absorption which energy corresponds to the energy required for the electron to transition from the top of the valence band to the bottom of the conduction band. Therefore, this absorption edge has been widely adopted to determine the forbidden band width. The optical absorption coefficient (*a*) and photon energy (*hv*) has a relation as such:

$$\alpha h\nu =A{\left(h\nu -E_{g}\right)}^{m}$$ (1)

Where *E*_g_ is the optical bandgap of the materials, *A* is a constant, and *m* hinges on the type of transition: *m* = 1/2 when band transition is indirect, and *m* = 2 when band transition is direct (Deng et al., 2013). Figure 4C delineates the linear relationship between (*ahv*)^2^ and *hv,* it indicates that the Bi_2_Se_3_ thin film has a direct bandgap structure. Extend the straight portion of the curve and the bandgap can be estimated as 0.47 eV. This value is a bit smaller than previously reported in the literature of 0.5 eV. Generally, the relationship between the forbidden band width and the carrier concentration can be expressed as such a linear relationship Δ*E* = Δ*E* (0) ‒ *kn* ^1/3^, where *n* is carrier concentration. During the growth of Bi_2_Se_3_ thin film, high-temperature annealing will cause the absence of Se atoms, which will create Se vacancies in the film, forming a natural *n*-type semiconductor. The bulk carrier concentration *n* increases, so the actual measured band gap is lower than the theoretical value. Figure 4D shows the infrared transmission spectrum of the Bi_2_Se_3_ thin film. The total transmittance of Bi_2_Se_3_ film in the wavelength range of 3.2 (3,125 wavenumber) −7.2 (1,400 wavenumber) μm is as high as 85% with no obvious characteristic absorption peak. The high transmittance (or low absorptivity), especially in the infrared band, is primarily attributed to the free carrier plasma edge around the far-infrared frequency. Due to the collective oscillation of conduction band electrons, called plasma oscillation, the transmittance of the film decreases at 7.2 μm suddenly. According to Drude’s free electron theory, the plasma oscillation frequency ω_p_ of the material determines the upper limit of the transmission wavelength. When the incident light frequency ω < ω_p_, the film exhibits strong reflectivity; when ω > ω_p_, the film exhibits transmittance. Thus, ω_p_ sets the low cutoff frequency of the transmission band.

### Electrical Characteristics of Bi_2_Se_3_ Thin Film

**FIGURE 5:**
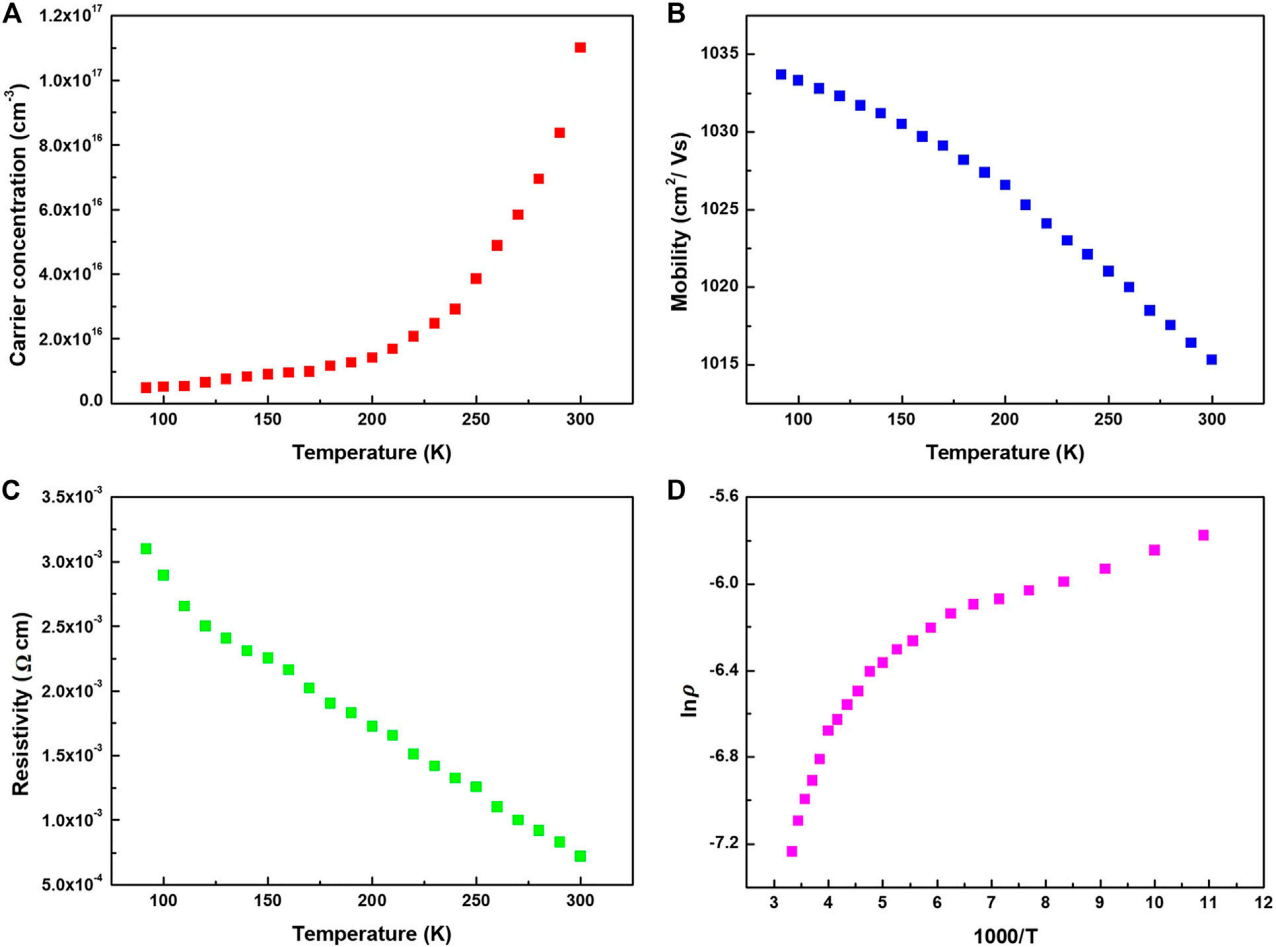
**(A)** Temperature dependence of: **(A)** Carrier concentration, **(B)** Hall mobility, **(C)** electrical resistivity **(D)** lnρ plotted as function of 1,000/T for the Bi_2_Se_3_ thin film.

In order to further analyze the electrical characteristics and transmission characteristics of Bi_2_Se_3_ thin films. Multiple temperature Hall effect measurement were carried out for the low temperature range. The influences of temperature on the resistivity, carrier density and mobility were investigated. As well as high transmittance in infrared band, the Bi_2_Se_3_ thin film also exhibits excellent electrical properties. The graphics of temperature dependent carrier concentration, Hall mobility and resistivity are shown in Figures 5A–C. The electron concentration of the film increases as the temperature rises, and the bulk electron concentration of the film at room temperature is about 1.15×10^17^cm^−3^. The Hall mobility decreases as the temperature rises, and the Hall mobility at room temperature is as high as 1,015 cm^2^/Vs, which is two orders of magnitude higher than the copper-iron ore series *p*-type infrared transparent conductive film we prepared previously. The reason for the high mobility is that Bi_2_Se_3_ belongs to a three-dimensional topological insulator structure. The basic feature of the structure is that there are four time-reversed symmetry points in the Brillouin zone of its surface state with Kramers degenerate phenomenon, which form the Dirac Cone. The apex of the Dirac cone is called the Dirac point, and the dispersion relationship between energy and momentum near the apex is linear. Due to the spin-coupling effect, the spin direction of the surface state is always perpendicular to the direction of momentum, so that electrons travel on the surface with low loss (or lossless) at a speed similar to the photon. The Hall coefficient of Bi_2_Se_3_ film is −7.8 cm^3^C^−1^ at room temperature, which indicates characteristic of n-type conduction. The room temperature conductivity of the film is 7 × 10^−4^ Ωcm. As shown in Figure 5D, Bi_2_Se_3_ film shows thermal activation behavior at room temperature, because the plots of ln*ρ* ∼1,000/*T* show a linear relation. The conductivity can be expressed by 1ρ=Aexp[−Ea/(kBT)] , where *A* is constant, *E*a is the thermal activation energy, and *k*B is Boltzmann constant. As temperature increases, electrical conductivity of the material increases, confirmative of the semiconductor nature of Bi_2_Se_3_ thin film in our study. The estimated *E*_a_ is 34 meV, which is less than 10% of the optical bandgap (E_g_ ≈ 0.47 eV) of Bi_2_Se_3_, indicating the electronic transport is thermally activated by a donor in the conduction band.

### Preparation and Measurement of Infrared Transparent Diode Device

**FIGURE 6:**
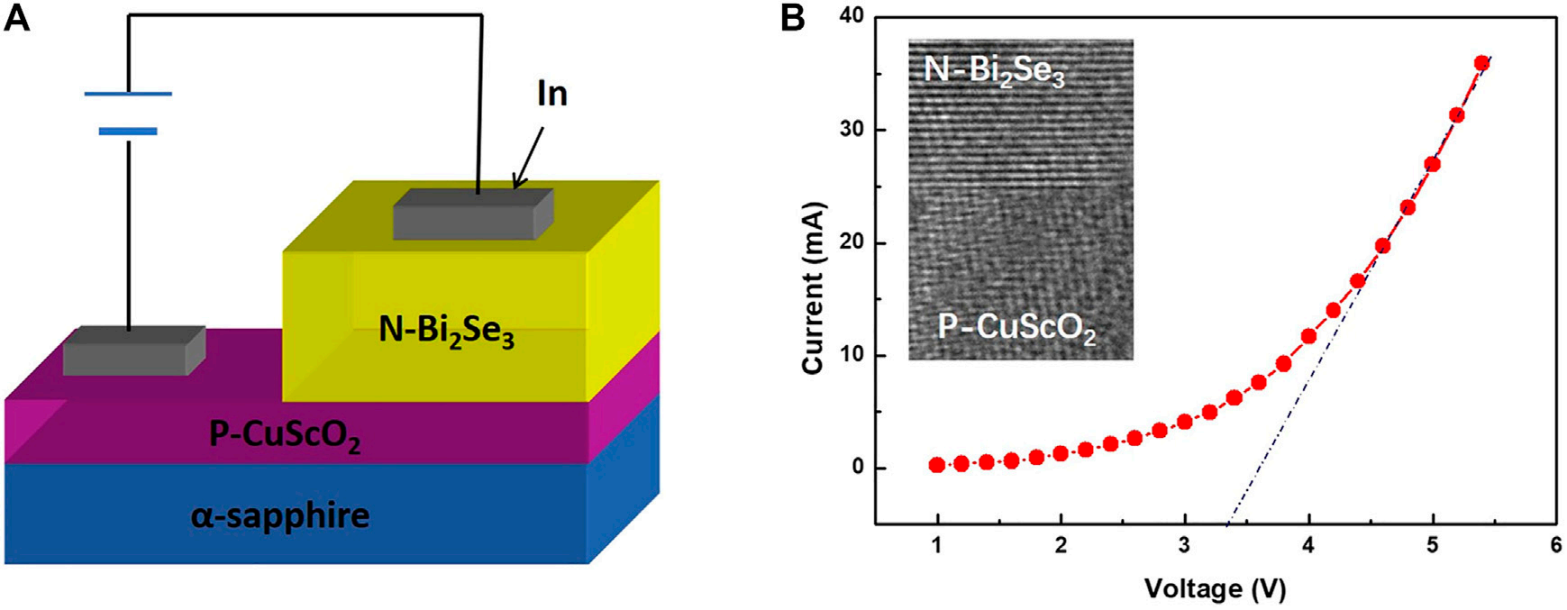
**(A)** Schematic structure of N-Bi_2_Se_3_/P-CuScO_2_ heterojunction diode device. **(B)** Current (*I*)—voltage (*V*) characteristics of N-Bi_2_Se_3_/P-CuScO_2_ diode device. The inset in **(B)** shows the cross-sectional HREM image of N-Bi_2_Se_3_/P-CuScO_2_ interface.

In order demonstrate the application of our thin film, we made N-Bi_2_Se_3_/P-CuScO_2_ infrared transparent heterojunction diode, the schematic diagram of which is shown in Figure 6A. The schematic diagram of this diode is shown in Figure 6A. First, we use the polymer-assisted deposition method to grow the CuScO_2_ film with a thickness of about 200 nm on a sapphire substrate, and then deposit a layer of Bi_2_Se_3_ thin film with a thickness of 150 nm by MBE. Finally, we evaporate In on the surface of the device as the electrode. Figure 6B shows the *I*–*V* curve of the heterojunction diode. It can be easily found the rectifying characteristic through the curve, accompanied by a threshold voltage of 3.3 V, which is consistent with the forbidden band width of CuScO_2_ (3.3–3.5 eV). Thus, hetero-epitaxial growth at the CuScO_2_ (lower part) and Bi_2_Se_3_ (upper part) interface is demonstrated.

## Conclusion

In this paper, we used molecular beam deposition (MBE) method to epitaxial grow Bi_2_Se_3_ thin film, which was then incorporated into N-Bi_2_Se_3_/P-CuScO_2_ infrared transparent heterojunction diodes. The unique growth and processing design of MBE underpins the high quality, epitaxial growth of the film. The Bi_2_Se_3_ thin film thus produced displayed remarkable optical transparency in IR region, and great *n*-type electrical conductivity. The CuScO_2_ film-based infrared transparent heterojunction diode has an abrupt interface, exhibits rectifying I—V characteristics with the threshold voltage of ∼3.3V. The results prove that the Bi_2_Se_3_ film has excellent optical and electrical properties in the wide-band infrared range. Such photoelectric properties make it a great candidate for window electrodes of infrared detectors, as well as other scenarios in the wide infrared wavelength range.

## Data Availability

The original contributions presented in the study are included in the article/Supplementary Material, further inquiries can be directed to the corresponding authors.

## References

[B1] Aydin E. De Bastiani M. Yang X. Sajjad M. Aljamaan F. Smirnov Y. (2019). Zr‐Doped Indium Oxide (IZRO) Transparent Electrodes for Perovskite‐Based Tandem Solar Cells. Adv. Funct. Mater. 29, 1901741. 10.1002/adfm.201901741

[B2] Chen T. c. Ma T. p. Barker R. C. (1983). Infrared Transparent and Electrically Conductive Thin Film of In2O3. Appl. Phys. Lett. 43 (10), 901–903. 10.1063/1.94199

[B3] Chuai Y.-H. Hu B. Li Y.-D. Shen H.-Z. Zheng C.-T. Wang Y.-D. (2015). Effect of Sn Substitution on the Structure, Morphology and Photoelectricity Properties of High C-axis Oriented CuFe1−xSnxO2 Thin Film. J. Alloys Compd. 627, 299–306. 10.1016/j.jallcom.2014.12.118

[B4] Chuai Y.-H. Shen H.-Z. Li Y.-D. Hu B. Zhang Y. Zheng C.-T. (2015). Epitaxial Growth of Highly Infrared-Transparent and Conductive CuScO2 Thin Film by Polymer-Assisted-Deposition Method. RSC Adv. 5, 49301–49307. 10.1039/c5ra07743e

[B5] Chuai Y.-H. Wang X. Shen H.-Z. Li Y.-D. Zheng C.-T. Wang Y.-D. (2016). Effects of Zn-Doping on Structure and Electrical Properties of P-type Conductive CuCr1−x Zn X O2 Delafossite Oxide. J. Mater. Sci. 51, 3592–3599. 10.1007/s10853-015-9679-4

[B6] Chuai Y. H. Bai Y. Zheng C. T. Liu C. Y. Wang X. Yue D. (2019). Chemical Modulation of Valence Band and Photoelectric Properties of Epitaxial P-type Infrared Transparent Conducting CuScO_2_ Thin Films. Mater. Res. Express 6, 126460. 10.1088/2053-1591/ab78c8

[B7] Chuai Y. Wang X. Zheng C. Zhang Y. Shen H. Wang Y. (2016). Highly Infrared-Transparent and P-type Conductive CuSc1−xSnxO2 Thin Films and a P-CuScO2:Sn/n-ZnO Heterojunction Fabricated by the Polymer-Assisted Deposition Method. RSC Adv. 6, 31726–31731. 10.1039/c6ra00919k

[B8] Deng Z. Fang X. Wu S. Zhao Y. Dong W. Shao J. (2013). Structure and Optoelectronic Properties of Mg-Doped CuFeO2 Thin Films Prepared by Sol-Gel Method. J. Alloys Compd. 577, 658–662. 10.1016/j.jallcom.2013.06.155

[B9] Hasan M. Z. Kane C. L. (2010). Colloquium: Topological Insulators. Rev. Mod. Phys. 82, 3045–3067. 10.1103/revmodphys.82.3045

[B10] Jain P. Nakabayashi Y. Haga K.-i. Tokumitsu E. (2020). Electrical Properties of In2O3 and ITO Thin Films Formed by Solution Process Using In(acac)3 Precursors. Jpn. J. Appl. Phys. 59, SCCB12. 10.7567/1347-4065/ab4a89

[B11] Jash A. Ghosh S. Bharathi A. Banerjee S. S. (2020). Coupling-decoupling of Conducting Topological Surface States in Thick Bi2Se3 Single Crystals. Phys. Rev. B 101, 165119. 10.1103/physrevb.101.165119

[B12] Johnson L. Moran M. (2001). “Infrared Transparent Conductive Oxides,” in SPIE Proceedings,Window and Dome Technologies and Materials VII,289 4375.

[B13] Khan A. Rahman F. Nongjai R. Asokan K. (2020). Structural, Optical and Electrical Transport Properties of Sn Doped In2O3. Solid State. Sci. 109, 106436. 10.1016/j.solidstatesciences.2020.106436

[B14] Lipkin N. Zipin H. Yadin Y. Klein Z. Dagan L. Marcovitch O. (2001). “Dual Band Transparent Conductive Coating,” in Proc. SPIE 4375, Window and Dome Technologies and Materials VII, 315 4375. 10.1117/12.439190

[B15] Liu F. Liu M. Liu A. Yang C. Chen C. Zhang C. (2015). The Effect of Temperature on Bi2Se3 Nanostructures Synthesized *via* Chemical Vapor Deposition. J. Mater. Sci. Mater. Electron. 26 (6), 3881–3886. 10.1007/s10854-015-2915-5

[B16] Park J. Y. Lee G.-H. Jo J. Cheng A. K. Yoon H. Watanabe K. (2016). Molecular Beam Epitaxial Growth and Electronic Transport Properties of High Quality Topological Insulator Bi 2 Se 3 Thin Films on Hexagonal boron Nitride. 2d Mater. 3, 035029. 10.1088/2053-1583/3/3/035029

[B17] Qi X.-L. Zhang S.-C. (2011). Topological Insulators and Superconductors. Rev. Mod. Phys. 83, 1057–1110. 10.1103/revmodphys.83.1057

[B18] Richardella A. Zhang D. M. Lee J. S. Koser A. Rench D. W. Yeats A. L. (2010). Coherent Heteroepitaxy of Bi2Se3 on GaAs (111)B. Appl. Phys. Lett. 97, 262104. 10.1063/1.3532845

[B19] Sizov F. Vuichyk M. Svezhentsova K. Tsybrii Z. Stariy S. Smolii M. (2021). CdTe Thin Films as Protective Surface Passivation to HgCdTe Layers for the IR and THz Detectors. Mater. Sci. Semiconductor Process. 124, 105577. 10.1016/j.mssp.2020.105577

[B20] Xia Y. Qian D. Hsieh D. Wray L. Pal A. Lin H. (2009). Observation of a Large-gap Topological-Insulator Class with a Single Dirac Cone on the Surface. Nat. Phys 5 (6), 398–402. 10.1038/nphys1274

[B21] Yang L. Han J. Zhu J. Zhu Y. Schlaberg H. I. (2013). Chemical Bonding and Optoelectrical Properties of Ruthenium Doped Yttrium Oxide Thin Films. Mater. Res. Bull. 48, 4486–4490. 10.1016/j.materresbull.2013.07.039

[B22] Zhang H. Liu C.-X. Qi X.-L. Dai X. Fang Z. Zhang S.-C. (2009). Topological Insulators in Bi2Se3, Bi2Te3 and Sb2Te3 with a Single Dirac Cone on the Surface. Nat. Phys 5 (6), 438–442. 10.1038/nphys1270

[B23] Zhang L. Wang B. Zhou Y. Wang C. Chen X. Zhang H. (2020). Synthesis Techniques, Optoelectronic Properties, and Broadband Photodetection of Thin‐Film Black Phosphorus. Adv. Opt. Mater. 8, 2000045. 10.1002/adom.202000045

[B24] Zhang W. Chen H. Ding R. (2021). Readout Integrated Circuit with Multi-Mode Background Suppression for Long Wavelength Infrared Focal Plane Arrays. Opt. Quant. Electron. 53, 4. 10.1007/s11082-020-02644-7

